# First Report of *Hemicriconemoides kanayaensis* (*Nematoda: Criconematidae*) on Tea Plantations in Iran

**DOI:** 10.2478/jofnem-2024-0044

**Published:** 2024-12-15

**Authors:** Negin Mirghasemi, Elena Fanelli, Alessio Vovlas, Alberto Troccoli, Salar Jamali, Francesca De Luca

**Affiliations:** Plant Protection Department, Faculty of Agricultural Sciences, University of Guilan, Rasht, Iran; Istituto per la Protezione Sostenibile delle Piante (IPSP), Consiglio Nazionale delle Ricerche (CNR), S. S. Bari, Via G. Amendola 122/D, 70126 Bari, Italy

**Keywords:** migratory nematode, *Camellia sinensis*, ribosomal DNA, mitochondrial COI, *Hemicriconemoides kanayaensis*

## Abstract

During a nematode survey in Iran, an abundant population of sheathoid, migratory, root-ectoparasitic nematodes was recovered from a tea, *Camellia sinensis* (L.), Kuntze plantation for the first time. Morphological and molecular characterization identified the Iranian population as *Hemicriconemoides kanayaensis*. The morphometrics of *H. kanayaensis* agreed with the original description. Phylogenetic relationships within *Hemicriconemoides*—based on ITS region, D2 to D3 expansion regions of the 28S rRNA, and the partial 18S rRNA genes along with the partial mitochondrial COI gene—confirmed the occurrence of *H. kanayaensis* on the tea plantation in Iran. Principal component analysis (PCA) confirmed the high intraspecific and interspecific variabilities among *Hemicriconemoides* species and between *H. kanayaensis* populations.

The genus *Hemicriconemoides* (Chitwood and Birchfield, 1957), often known as sheathoid nematodes, consists of migratory root-ectoparasitic nematodes. Currently, the genus consists of 55 valid species that have been reported all over the world in uncultivated and cultivated fields. Few *Hemicriconemoides* species have been implicated with the decline of many fruits, vegetables, and cash crops at high density worldwide (Inserra et al., 2014; Maria et al., 2019; Nguyen et al., 2020); most of them are not considered aggressive parasites. They are mostly distributed in temperate areas of the world, such as Africa, the Americas, Australia, South Asia, and South Europe.

Recently, several species of *Hemicriconemoides* have been also reported from different provinces in Iran: *H. phoenicis* on date palm (Azimi and Pedram, 2020), *H. californianus* (Pinochet and Raski, 1975) on faba bean (Azimi et al., 2016), *H. strictathecatus* on date palm (Eskandari et al., 2010; Van den Berg et al., 2015), *H. cocophilus* (Loos, 1949) on citrus and grapevine (Kheiri and Barooti, 1983), *H. mangiferae* (Siddiqi, 1961) on citrus (Kheiri and Barooti, 1983), and *H. chitwoodi* (Esser, 1960) on date palm (Jahanshahi et al., 1986). Tea production will increase in Iran in the upcoming years. Since tea plantations can be attacked by many nematode species, nematode surveys are routinely carried out (Luc et al., 2005; Mirghasemi et al., 2019). During a routine nematological survey of tea plantations in the Gilan province of Iran, a large population of *Hemicriconemoides* spp. was found in the rhizosphere of tea shrubs. Herein, the *Hemicriconemoides* population associated with *Camellia sinensis* was morphologically and molecularly characterized and identified as *Hemicriconemoides kanayaensis* (Nakasono and Ichinohe, 1961), recorded for the first time in Iran. *H. kanayaensis* is known as one of the main nematode tea pests in Japan (Nakasono and Ichinohe, 1961) and Taiwan (Germani and Anderson, 1991; Chen et al., 2007). Recently Maria et al. (2018) described three populations of *H. kanayaensis* from *Camellia grijsii* in China. Phylogenetic analyses based on ITS and D2 to D3 expansion domains of the 28S rRNA gene, the 18S rRNA gene, and the partial mitochondrial COI were also carried out. Principal component analysis (PCA) revealed large intraspecific and interspecific variations among *Hemicriconemoides* spp. independently from the collection sites of soil samples.

## Materials and Methods

### Nematode sampling

Soil samples were collected from the rhizosphere of tea plants in the Gilan province of Iran (37.136779 North and 50.076681 East) in March 2022. Nematodes were extracted from soil using the modified Baermann funnel method (Whitehead and Hemming, 1965; Southey, 1986). Sheathoid nematodes were the dominant species among samples and approximately 2000 nematodes/100 g soil were counted. For permanent slides, specimens were killed and fixed in hot aqueous 2% formaldehyde + 1% propionic acid, dehydrated in ethanol vapor, and mounted in dehydrated glycerin (Hooper, 1970). Light micrographs and measurements of specimens were taken with a Leica DFC 425 camera mounted on a Leica Diaplan (Wetzlar, Germany) compound microscope with incorporated software “Leica Microsystem®.” Morphological identification was based on the main diagnostic characters (Table 1).

**Table 1:** Morphometrics of *Hemicriconemoides kanayaensis* Nakasono & Ichinohe, 1961. female. All measurements are in μm and in the form: mean ± s.d. (range).

**Character**	**This study Iran**	**Maria et al., 2018 China**	**Nakasono & Ichinohe, 1961 Hangzhou Japan (Type Pop.)**	**Germani & Anderson, 1991 Taiwan**	**Chen et al., 2007 Taiwan**
**N**	10	15	20	12	*
**L**	494 ± 41.77 (409–560)	601 ± 43.2 (500–663)	571 (500–631)	510 (470–540)	(430–600)
**Rst**	24 ± 1.71 (21–27)	21.6 ± 1.0 (20.0–24.0)	-	-	-
**ROes**	35 ±1.89 (33–39)	30.4 ± 1.3 (29.0–33.0)	-	-	-
**Rex**	35 ± 2.2 (31–35)	33.2 ± 1.3 (31.0–36.0)	37 (30–44)	35 (31–38)	(35–41)
**Rv**	16.4 ± 14 (15–19)	15.1 ± 1.1 (14.0–17.0)	18 (16–21)	17 (16–18)	(13–19)
**Rvan**	5 ± 0.5 (5–6)	4.8 ± 0.7 (4.0–6.0)	-	-	-
**Ran**	12 ±1.7 (11–17)	10.3 ± 0.6 (9.0–11.0)	12 (11–15)	10 (8–11)	(9–13)
**a**	14.6 ± 1.3 (11.–16.8)	20.5 ± 1.9 (17.6–24.4)	21.5 (18.7–24.4)	17.3 (15.8–18.4)	(14.8–20.7)
**b**	4.6 ± 0.44 (3.9–5.2)	5.0 ± 0.3 (4.6–5.8)	4.8 (3.3–5.6)	4.8 (4.4–5.3)	(3.6–5.3)
**c**	14.5 ± 1.4 (13.6–16.6)	15.7 ± 1.2 (13.9–17.8)	14.3 (11.5–16.8)	12.9 (12–14.7)	(11.8–18.3)
**c’**	1.84 ± 0.18 (1.5–2.1)	2.0 ± 0.1 (1.8–2.3)	-	-	(1.5-2.5)
**m**	83.2 ± (81.3–85)	87.3 ± 1.3 (84.9–89.1)	-	-	-
**V**	89.1 ± 1.5 (88.4–90.7)	93.0 ± 0.6 (92.1–94.4)	88.9 (87.5–91.5)	88.3 (86.4–89.2)	(87.3–90.5)
**VL/VB**	2.26 ± 0.23 (1.95–2.7)	1.8 ± 0.1 (1.6–2.1)	-	-	-
**Stylet**	70.9 ± 4.6 (65.9–78.2)	76 ± 2.7 (72–82)	74 (66–79)	75 (66–78)	(69–79)
**ST%L**	6.9 ± 1.2 (6.2–7.1)	12.6 ± 0.9 (11.4–14.4)	-	-	-
**Stylet knob length**	2.6 ± 0.41 (2–3.3)	3.3 ± 0.4 (3.0–4.2)	-	-	-
**Stylet knob width**	6.83 ± 0.79 (6–8.8)	6.7 ± 0.4 (5.9–7.5)	-	-	-
**DGO**	5.4 ± 0.3 (5.1–5.9)	5.9 ± 0.5 (5.1–6.5)	-	-	-
**Pharynx**	107 ± 9.8 (104.5–116.3)	119 ± 6.3 (107–129)	-	-	-
**Anterior to excretory pore**	98.9 ±10.7 (88.3–116)	130 ± 6.4 (115–138)	-	-	(92–144)
**Max. body diam**	33.9 ± 1.7 (31–36)	29.5 ± 2.4 (26.0–34.0)	27 (22–29)	-	-
**Vulva body diam. (VD)**	22 ± 1.8 (20.7–25.5)	22.8 ± 1.3 (20.0–25.0)	-	-	(18–29)
**Vulva to tail tip**	49.9 ± 7.35 (42–69)	42 ± 3.0 (36–46)	-	-	-
**Anal body diam. (ABD)**	17.9 ± 1.59 (16–21.3)	19.3 ± 1.1 (17.0–21.5)	-	-	-
**Tail length (T)**	34.3 ± 4.16 (26.6–40)	38 ± 3.0 (33–46)	-	-	-

* Accumulative results of six populations from Taiwan.

### DNA extraction, PCR, and sequencing

Total DNA was extracted from individual nematodes as described by De Luca et al. (2004). The crude DNA was directly amplified. The ITS1–5.8S–ITS2 region was amplified from three specimens, using the forward primer 18Sext (5′ - TGATTACGTCCCTGC CTTT - 3′) and the reverse primer 26Sext (5′ - TTTCACTCGCCGTTACTAAGG - 3′) (Vrain et al., 1992); the mitochondrial COI from five specimens, was amplified using COI (5′ -GATTTTTTGGKCATCCWGARG- 3′) and XIPHR2 (5′ -.GTACATAATGAAAATGTGC CAC - 3′) (Lazarova et al., 2006); D2A to D3B expansion segments of the 28S rRNA gene from two specimens was amplified using the primers D2A (5′ - ACAAGTACCGTGGGGAAAGTTG - 3′) and D3B (5′ - TCGGAAGGAACCAGCTACTA - 3′) (Nunn, 1992); the 18S rDNA was amplified from two specimens, using the 18SnF (5′ - TGGATAAC TGTGGTAATTCTAG AGC - 3′) and 18SnR (5′ - TTACGACTTTTGCCCG GTTC - 3′) (Kanzaki and Futai, 2002). The following PCR cycling conditions were used for ribosomal amplification: an initial denaturation at 94°C for 5 min; 35 cycles of denaturation at 94°C for 50 sec, annealing at 55°C for 50 sec; and extension at 72°C for 1 min and a final step at 72°C for 7 min.

These were the conditions for mitochondrial COI amplification: an initial denaturation at 94°C for 5 min; 35 cycles of denaturation at 94°C for 40 sec, annealing at 48°C for 40 sec; extension at 72°C for 1 min; and a final step at 72°C for 7 min. The PCR products were separated in 1% agarose gel in a TBE buffer (40 mM Tris, 40 mM boric acid, and one mM EDTA) for assessment of the DNA bands. Purified COI fragments of three specimens were eluted from the gel and cloned in a TA cloning vector (Invitrogen). Ten clones were sent for sequencing to MWG Eurofins (Germany).

### Phylogenetic analysis

BLAST search at NCBI was performed using all new sequences obtained to identify the corresponding and closest sequences to *H. kanayaensis*. Multi-alignment was performed using the computer program MAFFT v. 7 software (Katoh et al., 2019). BioEdit was used for sequence alignments, which were edited manually in order to improve the multi-alignment. The best-fitted models of nucleotide substitution using the phylogenetic analysis were selected using jModelTest v. 2.1.10 (Darriba et al., 2012) with the Akaike information criterion (AIC). The Akaike-supported model, base frequency, proportion of invariable sites, and gamma distribution shape parameters and substitution rates in the AIC were then used in the phylogenetic analyses. MrBayes 3.1.2 (Ronquist and Huelsenbeck, 2003) was used to produce Bayesian phylogenetic reconstructions of the data sets. The General Time Reversible substitution model with gamma distributed rate variation across sites (GTR+G) was used as the optimal nucleotide substitution model for the analyses. The Bayesian analysis was initiated with a random starting tree, and the Markov chain Monte Carlo algorithms were set to four with 2 × 10^6^ generations samplings at intervals of 100 (Larget and Simon, 1999). Two runs were performed for each analysis. After discarding burning samples and evaluating convergence, the remaining samples were retained for further analyses. The topologies were used to generate a 50% majority rule consensus tree. Posterior probabilities are given on appropriate clades. Trees from all analyses were visualized using FigTree software version v. 1.42 and edited with GIMP 2.8.14. The tree was visualized and saved with FigTree 1.4.4 (Rambaut, 2018).

### Multivariate morphometric analysis

To evaluate the degree of morphological variations within *Hemicriconemoides* species, including those of the present investigation, PCA of different morphological traits was conducted (Archidona-Yuste et al., 2016; Khan et al., 2019; Sharma and Chaubey, 2023). PCA was carried out in XLSTAT (Addinsoft, 2007). Measurements were obtained from literature, using the mean values for each population (Supplementary Table 1) and normalized through XLSTAT prior to their analyses. Eleven diagnostic characters were used: body length (L), stylet length (ST), percentage distance from anterior end to vulva/body length (V), total number of body annules (R), annules from anus to tail terminus (Ran), annules from vulva to tail terminus (RV), annules from anterior extremity to excretory pore (Rex), and “de Man’s indices” “a”, “b” and “c.” The score values for the first two components were determined to form a two-dimensional plot (PC1 and PC2) for each population, based on factor loadings given by the software.

## Results

### Morphological characterization

Measurements of the Iranian *H. kanayaensis* population from Iran (Table 1) agreed with the measurements of the original population from Asiatic countries (Nakasono and Ichinohe, 1961; Germani and Anderson, 1991; Chen et al., 2007; Maria et al., 2018).

#### Female

Specimens of *H. kanayaensis* were characterized by cylindrical bodies that are slightly arcuate ventrally after heat killed. The body was covered with a cuticular sheath that was loosely separated from the anterior body and attached to the posterior part of the body. The sheath annules of the body were coarse and rounded, without appendages. The first annulus of the lip region was rounded at the outer edges and set off by a constriction. The side view face of the six sectors of the lip regions was observed easily and lateral edges of labial annulus were irregular and distinctly wider than the submedian four. The basal plate of the labial framework was vigorously sclerotized. The stylet was strong and straight or slightly curved with vigorous basal knobs that had margins directed anteriorly. The excretory pore was detected at the anterior end, between the 33^rd^ to 38^th^ annule of the body. The hemizonid and hemizonion were absent. The vulva was posterior, straight, located 15 to 19 annuli from the terminus, and 88.4% to 90.4% of body length. The spermatheca was spherical-shaped and filled with numerous rounded sperm cells that are commonly located on the left side of the end of the uterus. This nematode had a single ovary and was prodelphic. The anus was small, distinct, observed easily, and situated on the 11^th^ to 17^th^ annuli from the posterior terminus of the body. The tail’s shape was elongated and widely conoid with a smoothly rounded tip.

#### Males

Males were present. The cuticular sheath was absent, and lateral fields with four smooth lines were present. The spicule was slightly curved, with 20.5 μm to 24.6 μm long protruding from the cloaca. Gubernaculum was 4.8 μm long, and the bursa was absent. The shape of the tail was an elongated terminus conoid (Table 2).

**Table 2:** Morphometric data of male of *Hemicriconemoides kanayaensis*. All measurements are in μm and in the form: mean ± s.d.

**Character**	**This study, Iran**	**Chen et al. 2007, Taiwan (Pinglin)**	**Chen et al. 2007, Taiwan (Rueisuei)**	**Nakasono & Ichinohe 1961, Japan**
**N**	8	8	8	7
**L**	437 ± 29 (396–473)	420 ± 10 (400–440)	430 ± 30 (400–460)	457 (422–489)
**a**	27.6 ± 1.7 (25.9–29.5)	28.9 ± 2.9 (24.7–33.9)	29.8 ± 2.3 (26.7–33.9)	29.7–32.6
**c**	15 ± 1.06 (13.9–16.6)	15.5 ± 1.1 (13.8–17.5)	16.4 ± 1.2 (14.8–17.9)	14.6 (14.6–15.1)
**c′**	2.37 ± 0.26 (2.14–2.61)	2.6 ± 0.2 (2.2–2.8)	2.6 ± 0.2 (2.1–2.7)	-
**EP**		91 ± 7 (83–100)	99 ± 5 (92–107)	86
**Max. body diam**	15.9 ± 1.24 (14.6–17.7)	-	-	-
**Anal body diam. (ABD)**	12.5 ± 1.16 (11.5–13.6)	10 ± 1 (10–11)	10 ± 1 (10–11)	-
**Tail length (T)**	29.3 ± 2.74 (26.2–33)	27 ± 2 (24–31)	26 ± 2 (23–29)	-
**Spicule**	23.1 ± 1.81 (20.5–24.6)	26.5 (n=4) (25.7–26.7)	25.4 ± 1.1 (24.2–27.0)	23.8
**Gubernaculum**	4.83 ± 1.16 (3.7–6.9)	-	-	-

#### Juveniles

The cuticular sheath was absent in juveniles. The body was smooth and curved ventrally. The lip region was smooth and rounded, continuous with the body. No lateral field was visible. The juvenile population measured as follows (n = 5): L = 0.222 mm, a = 12.4; b = 2.9; c = 40.8; stylet = 35.7 μm; R = 127; Rex = 40; Ran = 5.

#### Remarks

*Hemocriconemoides kanayaensis* has been reported in Japan, China, and Taiwan. This is the first report of *H. kanayaensis* in an Iranian tea plantation. The Iranian specimens were morphologically and morphometrically in agreement with the original description and the populations from Taiwan, but small differences regarding a shorter body length of the Iranian population compared to the Chinese population: 494 μm (409 to 560 μm) versus 601 μm (500 to 663 μm), a shorter stylet of 71 μm (65.9 to 78.2 μm) versus 76 μm (72 to 82 μm), and a lower V value of 89 μm (88.4 to 90.7 μm) versus 93 μm (92.1 to 94.4 μm). Intraspecific variabilities could be due to different geographical origins.

*H. kanayaensis* is morphological like *H. strictathecatus* (Esser, 1960) and *H. mangiferae* (Siddiqi, 1961). *H. strictathecatus* differed by having rounded stylet knobs, and the first annulus of the lip region was directed outward and disc-shaped (Nakasono and Ichinohe, 1961). *H. mangiferae* is clearly distinguished by the shape of the first annulus of the lip region, which is angular, and the character of “c” in the female is smaller than in *H. kanayaensis.* Moreover, the lip region of the male of *H. mangiferae* has five annules without a protruded first annulus and the bursa.

#### PCA

The PCA based on morphometric data showed that the four populations of *H. kanayaensis* are characterized by high intraspecific variability. Moreover, the Iranian population confirmed little variations with *H. strictathecatus* and *H. mangiferae*. The PCA based on the morphometry of females showed an accumulated variability of 59.1% (Fig. 2). The contribution of PC1 and PC2 was found out to be 38.4% and 20.7%, respectively (Fig. 2). Four parameters — b, c, V, and R — were negatively correlated across nematode per species in PC1. Ten out of 14 characters were positively correlated across isolates, and the remaining characters were negatively correlated considering PC1 (Fig. 2). The highest coefficient of correlation was observed in annuli from the anterior end to the excretory pore (r = 0.42) and stylet (r = 0.41) in PC1. Considering PC2, eight out of 11 characters were positively correlated, while the remaining were negatively correlated (Supplementary Table 1). The highest coefficient of correlation was observed in b (r = 0.51) in PC2.

**Figure 1:**
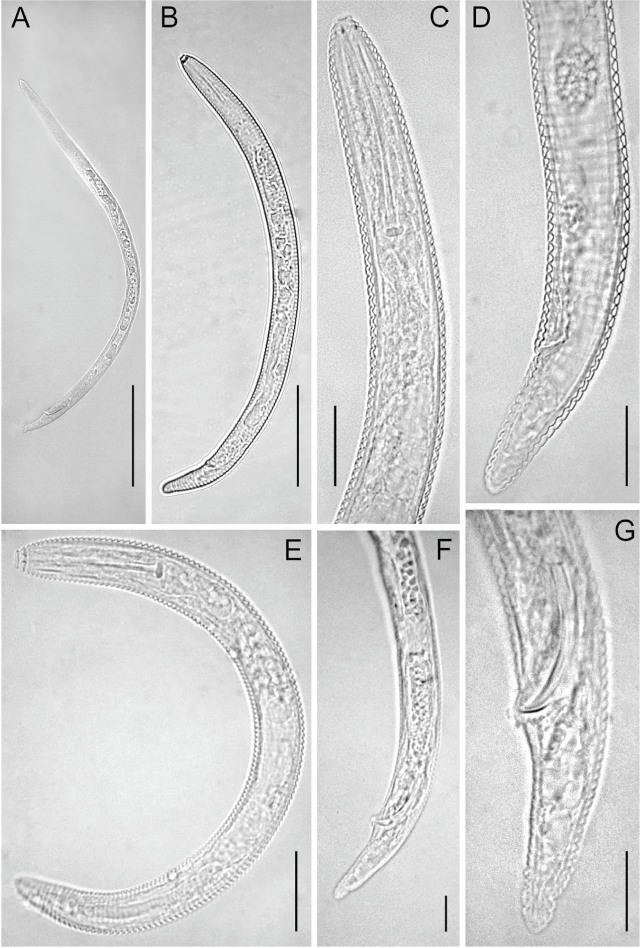
Light photomicrographs of *Hemicriconemoides kanayensis*. A: Male entire body; B: Female entire body. C: Female pharyngeal region; D: Female posterior region; E: Juvenile stage entire body; F-G: Male tail with spicules. (Scale bars: A, B = 100 μm; C-E, G = 20 μm, F = 50 μm).

**Figure 2:**
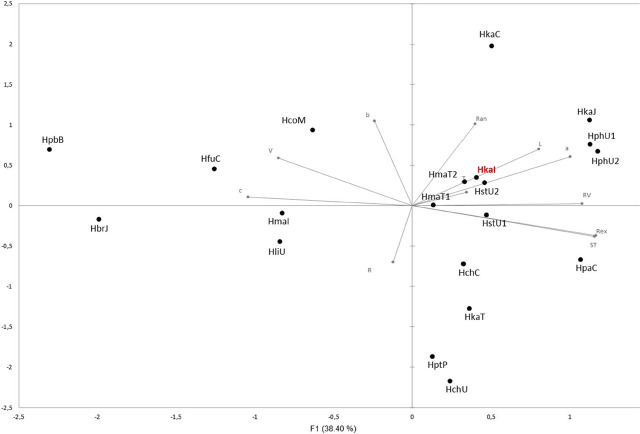
Biplot of principal component analysis (PCA) based on of the morphometric characters of *H. kanayensis* from Iran (HkaI), compared with population previous descripted in literature: *H. kanayensis* from Japan (HkaJ), from Taiwan (HkaT); *H. mangiferae* from Taiwan (HmaT1 and HmaT2) and from India (HmaI); *H. litchi* from USA (HliU); *H. cocophilus* (HcoM) from Mozambique; *H. chitwoodi* from China (HchC) and from USA (HchU); *H. paracamelliae* (HpaC) from China; *H. brachyurus* (HbrJ) from Japan; *H. pseudobrachyurus* (HpbB) form Belgium; *H. phoenicis* (HphU1 and HphU2) from USA; *H. parataiwanensis* (HptP) from Papua New Guinea; *H. fujianensis* (HfuC) from China; *H. strictathecatus* from USA (HstU1 and HstU2).

#### Molecular analysis

The amplification of ITS, the 18S rRNA gene, the D2 to D3 expansion domains of the 28S rRNA gene, and the COI were conducted on individual nematodes of *H. kanayaensis*, yielding single fragments of 1043 bp, 1618 bp, 748 bp, and 435 bp, respectively. Two amplified products for ITS, the 18S rRNA gene, and the D2 to D3 expansion domains of the 28S rRNA were directly sequenced. The ITS sequences of Iranian *H. kanayaensis* were identical and showed a 99.3% to 99.7% (a difference of 2 bp to 5 bp) identity to *H. kanayaensis* from the database. Very few ITS sequences of *H. kanayaensis* are present in the database from China and Taiwan. Fourty-nine sequences of *Hemicriconemoides* spp. and one sequence of *Paratylenchus aculenta*, as the outgroup, were aligned.

The D2 to D3 expansion domains of the Iranian *H. kanayaensis* showed a 99.4% to 99.7% identity with the corresponding sequences of Chinese *H. kanayaensis* populations (1 to 4 different nucleotides) present in the database. Thirty-nine sequences of *Hemicriconemoides* spp. along with the new Iranian *H. kanayaensis* sequence were aligned.

The 18S rRNA sequences showed a 99.25% to 99.5% identity (a difference of 5 bp to 12 bp) with *H. kanayaensis* from China and a 98% similarity with other *Hemicriconemoides* species present in the database. A total of 31 sequences of *Hemicriconemoides*, including the new one, were aligned, and *A. agricola* was used as the outgroup.

**Figure 3:**
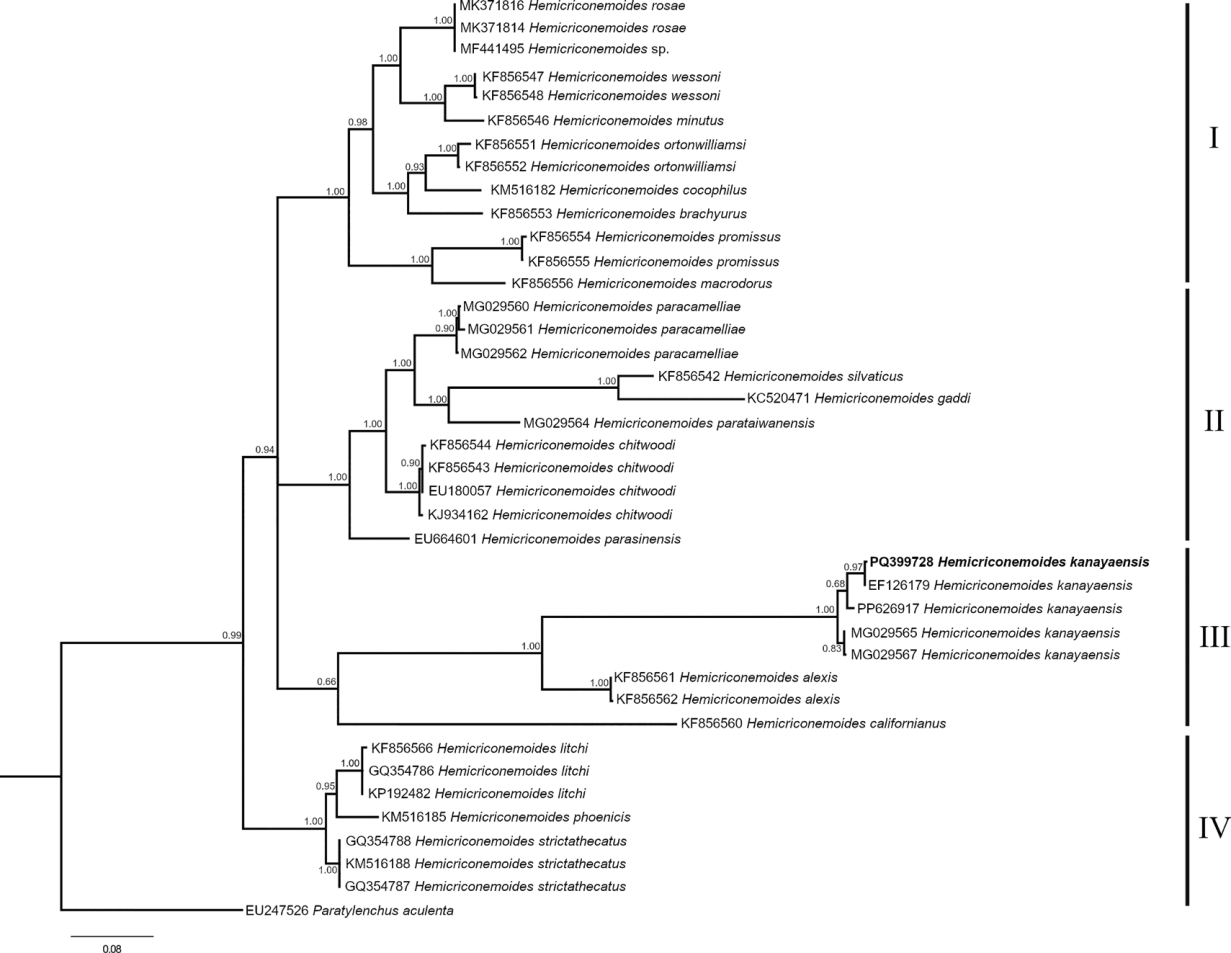
Phylogenetic tree of ITS of *Hemicriconemoides kanayaensis* and *Hemicriconemoides* species. Bayesian 50% majority rule consensus tree as inferred from ITS sequence alignment under General Time Reversible (GTR) model across lineages along with a gamma (I+G) distributed rates across sites. Posterior probabilities greater than 0.50 are given for appropriate clades. Newly obtained sequences in this study are shown in bold. Scale bar = expected changes per site.

**Figure 4:**
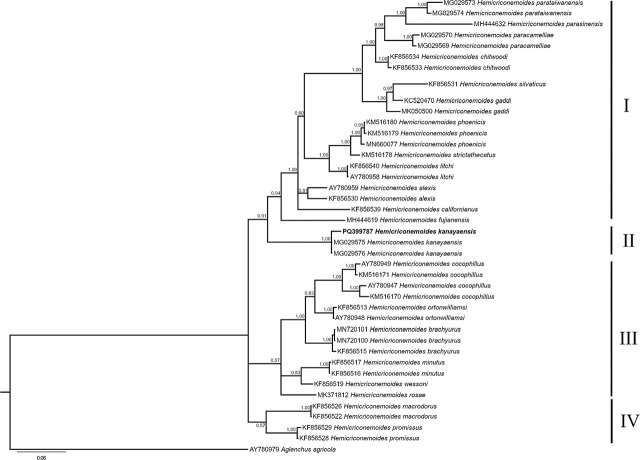
Phylogenetic tree based on D2 to D3 expansion domains of the 28S rRNA gene of *Hemicriconemoides kanayaensis* and *Hemicriconemoides* species. Bayesian 50% majority rule consensus tree as inferred from D2-D3 sequence alignment under General Time Reversible model across lineages along with a gamma distributed rates across sites (GTR+G). Posterior probabilities greater than 0.50 are given for appropriate clades. Newly obtained sequences in this study are shown in bold. Scale bar = expected changes per site.

The phylogenetic trees, ITS, D2-D3 and 18S, showed four main clades and only those of ITS and D2-D3 are shown (Figs. 3,4; Supplementary Fig. 1). The Iranian *H. kanayaensis* sequences, obtained in the current study, grouped with the corresponding sequences of *H. kanayaensis* from China (99% support), and the group was located at basal position of clades I and II.

#### Mitochondrial COI

**Figure 5:**
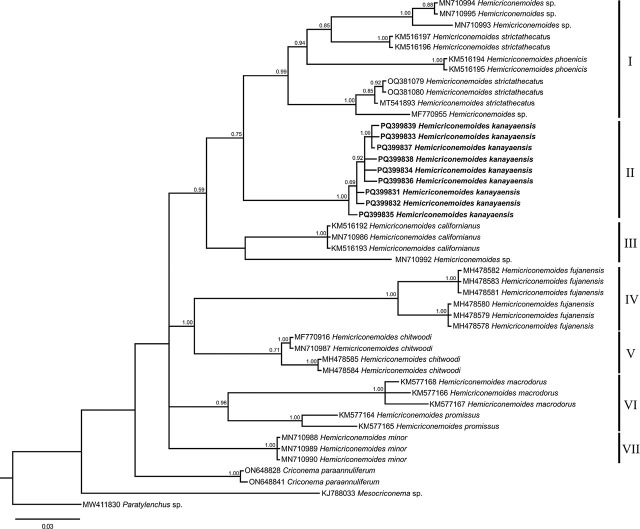
Phylogenetic tree based on Mitochondrial COI of *Hemicriconemoides kanayaensis* and *Hemicriconemoides* species. Bayesian 50% majority rule consensus tree as inferred from COI sequence alignment under General Time Reversible model across lineages along with a gamma distributed rates across sites (GTR+G). Posterior probabilities greater than 0.50 are given for appropriate clades. Newly obtained sequences in this study are shown in bold. Scale bar = expected changes per site.

The COI of three individual specimens were cloned and sequenced. Ten new COI sequences of *H. kanayaensis* were obtained in this study, and the intrapopulation sequence diversity was very low, from 0% to 1.1% (0 to 5 nucleotides). No corresponding sequences were present in the database for the COI gene of *H. kanayaensis*; the closest sequences were those of *H. strictathecatus*, showing 90% similarity. Pairwise, distances between Iranian *H. kanayaensis* with other *Hemicriconemoides* varied from 8% to 18% (35 to 79 nucleotides). The phylogenetic analysis based on COI grouped all newly obtained sequences of *H. kanayaensis* with *H. strictathecatus* and *H. phoenicis*, with 100% support and short branch differences suggesting intrapopulation variability (Fig. 5).

## Discussion

The present study reports on the occurrence of *H. kanayaensis* on tea in Iran for the first time. *H. kanayaensis* causes serious damage to tea plantations (Chen et al., 2007; Inserra et al., 2014; Maria et al., 2018) in Japan and in China. Thus, its occurrence in Iran could represent an alarming sign for the economy, as tea is the main beverage in Iran. The occurrence of *H. kanayaensis* in Iran extends the geographical distribution of this species in Asia.

The morphology (Fig. 1) and the morphometrics (Table 1) of Iranian *H. kanayaensis* show higher intraspecific differences compared with the original species description (Nakasono and Ichinohe, 1961) and the recent descriptions. This finding confirmed the previous investigations reporting that *Hemicriconemoides* spp. are characterized by high intraspecific morphological variability, independently from the collection sites of soil samples. PCA also confirmed high intraspecific variability of *H. kanayaensis* populations and the positive correlation between body length and a ratio (Fig. 2) with *H. strictathecatus*, while molecularly they were in different subgroupings.

Thus, an integrated approach — combining morphological, molecular, and principal component analyses — was used to characterize the *H. kanayaensis* population in tea plantation from Iran. The phylogenetic analyses based on the ITS, 28S rRNA, and 18S rRNA genes are congruent with each other, supporting the grouping of all populations of *H. kanayaensis* and their close relationships with Clade I and II as reported by other authors (Figs 3,4; Supplementary Fig. 1).

As *H. kanayaensis* is known as a pathogen for tea, a more extensive survey should be undertaken in the major Iranian tea-growing areas to understand the role that this nematode is playing in Iranian tea plantations and to prevent its spread.

## Supplementary Figure

**Supplementary material Table 1:** Correlations between variables and components.

	**F1**	**F2**	**F3**	**F4**	**F5**	**F6**	**F7**	**F8**	**F9**	**F10**	**F11**
L	0,290	0,345	0,238	−0,187	−0,459	−0,314	−0,165	−0,296	−0,184	−0,102	0,486
a	0,362	0,298	0,126	−0,431	0,107	−0,072	0,090	0,265	0,097	−0,499	−0,475
b	−0,086	0,515	−0,249	−0,260	−0,288	0,454	−0,063	−0,064	0,349	0,408	−0,113
c	−0,376	0,053	0,223	−0,220	0,356	−0,413	−0,466	−0,208	0,439	0,071	−0,041
V	−0,307	0,290	0,421	−0,066	0,130	−0,042	0,371	0,574	−0,022	0,184	0,347
R	−0,043	−0,343	0,415	−0,508	0,100	0,552	−0,185	−0,136	−0,278	0,018	0,075
Rex	0,421	−0,182	0,105	−0,071	0,245	0,048	0,502	−0,317	0,528	0,107	0,261
RV	0,390	0,012	−0,319	−0,002	0,334	0,140	−0,484	0,421	0,085	−0,008	0,443
Ran	0,144	0,497	0,078	0,234	0,574	0,096	0,023	−0,348	−0,413	0,170	−0,108
ST	0,418	−0,188	0,230	−0,031	−0,113	−0,279	−0,126	0,214	−0,096	0,681	−0,333
T	0,125	0,082	0,543	0,584	−0,166	0,321	−0,264	0,053	0,314	−0,175	−0,097
